# Foreign

**Published:** 1840-08-01

**Authors:** 


### FOREIGN.

*Cases of Tubercular Meningitis.* By P. Hennis Green, M. D.

Case 1.—Lefere, æt. six years, admitted into the Children's Hospital, Paris, Sept. 14th, 1835. Fine, healthy looking child; father scrofulous, mother healthy. The child had been brought home from nurse at the age of four years, and since then his parents had remarked that he was disinclined to play or move about. In July and August he was slightly indisposed, coughed a little, and occasionally was unable to pronounce words distinctly. Sept. 11th. The child was frightened, and soon after vomited; this recurred on the 12th. No active treatment had been employed. Sept. 13th. Somnolence, which continued up to the time of admission; face alternately pale and flushed; obstinate constipation.

Sept. 16th. Deep coma; face flushed; skin rather warm; no peculiar heat of forehead, or pulsation of temporal or carotid arteries; eyelids closed, but easily opened; pupils natural, slightly moveable; no strabismus; eyelids occasionally convulsed; sight and hearing completely lost. Mouth cannot be opened from a rigidity of the muscles; deglutition difficult; both arms somewhat stiff, with automatic motions of the fingers of the left hand; lower extremities relaxed; muscles of neck and back extremely rigid. Sensibility of right arm completely lost, left nearly so. Pulse full, irregular, slow, 64. Respiration 18.24, sonorous. Had one evacuation yesterday, and urinated two or three times. Abdomen retracted and free from pain.

Half an hour afterwards, the face was of a bright scarlet tint, which passed rapidly away, leaving a stern frown on the countenance; the trismus gave way, allowing an examination of the tongue, which was moist and clean. The child opens his eyes, but cannot see; eyeballs insensible to touch. Left arm now relaxed; lower extremities sensible. Tisane; injection; sinapisms to legs.

Sept. 17th. Insensible as yesterday; eyelids sometimes closed, sometimes half open; eyeballs roll obliquely upwards and outwards; pupils contracted, slightly oscillating; no strabismus; colour and expression natural; appearance of deep sleep. Lips clean and dry; no trismus; breath foul; upper extremities relaxed; abdomen greatly retracted; back and neck less stiff than yesterday. On raising the patient the face becomes of a bright scarlet, and the pulse small, quick, and irregular; pulse, ordinarily, full and regular, 108. Respiration 22, sighing; skin cool; no unnatural pulsation of carotids, &c. Face very red, with alternations of paleness. One hard evacuation from injection.

M. Guersent remarked, that as the most active treatment in acute meningitis had almost invariably failed, he would now do what he had never before dared to do, and leave this case to *nature*. He would thus see how long the child would hold out, and perhaps learn how far death had been accelerated by the remedies commonly employed; the longest case that he had ever seen, lasted only thirty-one days.

The prescription, therefore, was dog-grass, oxymel, injection of milk.

Sept. 18th. Same coma; eyelids wide open; expression natural; pupils contracted and dilated alternately; skin slightly warm; pulse regular, 120; respiration 30. On motion, the blood rushes to the face, and the child sighs. No convulsions, no moaning or crying; sight and hearing lost; left arm a little sensible, and when pinched the fingers move; but neither the muscles of the arm or face, give any signs of consciousness; right arm completely paralyzed; lower extremeties paralyzed. Lips clean; abdomen not painful, retracted. From injection, one copious solid stool. Continue treatment.

Sept. 19th. Patient dying; face dull pale colour; eyelids open; eyes much injected, occasionally turned up; pupils contracted and dilated; respiration embarrassed, with tracheal rattle 50; pulse nearly imperceptible; no convulsions; extremities all paralyzed, but soles of feet strongly arched, resisting extension; fingers flexed, not stiff; a few sudamina; skin very warm and moist. At six o'clock, the child swallowed fluids, but with difficulty; he died 20 minutes after the visit.

*Post mortem Examination* 24 hours after death. Anterior surface of the body and face, pale; posterior marked with blue patches; arms relaxed, fingers flexed and stiff; nails blue; lower extremities very rigid; toes and soles of feet strongly flexed as before death.

*Head.*—No fluid in the great arachnoid cavity, but on removing the brain about two ounces of serum tinged with blood was found at the base of the cranium, (escaped probably from the ventricles.) Arachnoid covering brain moist; vessels ascending from base of brain deeply congested with dark blood; most marked on the left side.

*Left Hemisphere.*—Here, opposite the antero-superior edge of the ear, there is a patch of yellow infiltrated matter, which extends with the pia mater between the anterior and middle lobes to the commissure of the optic nerves; there is also a layer of yellow lardaceous matter which ascends for about two inches into the left fissure of Sylvius; here the pia mater is considerably thickened, and contains a number of yellow granulations, either mixed with, or separate from, the larger mass. It is also closely adherent to the cortical substance, which cannot be removed without being broken down by the handel of the scalpel; this portion of the brain is of a pink colour and softened. The lardaceous matter varies considerably in thickness; at one point it is one quarter of an inch thick, and bears a close resemblance to the scrofulous matter deposited under the pleura. The pia matter, where it can be distinguished between the granulations, is much injected. Between the convolutions in the neighborhood of the lardaceous matter, there are numerous granulations which, at first sight, might be taken for air-bubbles; to each granule the cortical substance is adherent. Nearly in the centre of the surface of this hemisphere, there is a broad patch of sero-sanguineous effusion in the pia mater, and below this again, a patch of granulations, the cellular membrane being highly injected, thickened, and adherent to the brain. The substance of this hemisphere was minutely divided, but no trace of tubercles were discovered in its anterior.

*Right Hemisphere.*—Nearly the whole surface is covered with an irregular sero-sanguineous effusion, which is very dark in some places. On the centre, there are two or three large isolated granulations, along the track of one of the vessels; between the convolutions in the immediate neighbourhood of these tubercles, the pia mater is deeply injected, but not adherent to the cortical substance, although the latter is softened; the cellular membrane into which the sero-sanguinolent fluid is effused, is very tender and easily detached from the brain. No tubercles in the substance of this hemisphere; medullary matter much injected, and the pia mater which dips in among the convolutions, forms so many deep red lines. Plexus choroides healthy; lateral ventricles rather enlarged, containing a small quantity of reddish serum; septum lucidum, fornix, and parietes of ventricles, firm. Membranes of cerebellum healthy, but there are a few granulations on its lower surface. Pons Varolii normal.

Spinal marrow healthy; posterior vessels, however, more injected than anterior. Tubercles in lungs and bronchial glands; in the liver, and externally; also in the spleen.

Mucous membrane of stomach slightly injected and softened; small intestines healthy, large mucous membrane deep purple and softened; kidneys, &c. healthy; no tubercles in mesenteric glands.

Case 2.—Bouillett, æt. four years, admitted Aug. 30th, 1835. Mother died of phthisis at the age of 32; father very subject to coughs. The boy enjoyed good health, and was gay, until three months ago, when his mother died; he was then suddenly seized with convulsions, which lasted three hours, but did not recur nor leave any unfavourable symptom. Aug. 25th. Suddenly seized with headach, became dull, and vomited for five days in succession. The second day confined to bed, constantly complaining of his head, and placing his hand on his temples; also of pain in abdomen. Constipation.

On admission, the child complained of his head; occasional drowsiness; pulse irregular, 72; great thirst. Cold lotions to head; sinapisms to legs and feet; purgative injection.

Aug. 31st. Child lies on his side with knees drawn up; uneasy, turning from side to side; face pale; lips dry; tongue moist and furred; eyes natural; no strabismus; no pain in head; sighs very frequently; skin slightly warm; pulse 74, irregular; respiration slow, 10, sighing; intolerance of light; no vomiting since admission; deglutition easy; no thirst; no lesion of sensibility or motility; four stools. Six leeches to temples; cold lotions to forehead; injection.

Sept. 1st. Symptoms as yesterday; occasional wandering and crying during the night. A current of cold water was kept constantly flowing on the crown of the head.

Sept. 2d. Patient apparently comatose, yet when spoken to opens his eyes and answers in a clear, slow voice; face cool; forehead warm, although the cold water runs constantly over the head; no headach, but light painful; constantly carries hand to head; right eyelid contracts strongly when raised by the finger, left offers no resistance; pupils sensible, sometimes contracted, sometimes a little dilated. Respiration 20, sighing, irregular; pulse 72.78, irregular. No changes of colour, no convulsions; rigidity of body and limbs; sensibility of limbs unimpaired; fits of laughter yesterday alternating with delirium and sharp cries; no pain in abdomen on pressure; one dark offensive dejection; urine involuntary; castor oil, half oz.; emollient injection; oxymel.

Sept. 3d. Quiet as yesterday; strabismus now evident; pupils not dilated; sees well; forehead very warm, but no pulsation of the arteries; no pain in head, only about the eyes, (no reliance, however, can be placed on the child's answers;) delirium last night; now answers distinctly but slowly; no convulsions; sensibility of face acute, that of extremities diminished. No vomiting; tongue moist and white, not trembling, (the peculiar trembling of the tongue when projected, in typhus, never occurs in hydrocephalus;) pulse 80, irregular in force and rythm; respiration twenty-four, slightly sighing. The boy now carries his hand to his head and complains of headach. Repeat castor oil; continue irrigation.

Sept. 4th. As yesterday; pulse small, 140; respiration 26, irregular; has passed two yellow fluid stools. Continue treatment; the water which constantly falls on the head from the height of one foot is of the temperature of the room.

Sept. 5th. Evidently more feeble; face pale and sunken; voice feeble, answers extremely slow; sighing and plaintive moans from time to time; squinting continues; pupils largely dilated; whenever he is moved the body continues to become stiff, and the arms now slightly resist extension; no paralysis yet. Skin cool; pulse very feeble, 156; respiration 26; no convulsions; abdomen excessively retracted; one fluid stool. When asked to eat, said he should like an egg. Before quitting the hospital, I again examined the child, found the skin quite cold, the pulse insensible, and the neck perfectly rigid; he seemed to be at the point of death.

Sept. 6th. Moaning and crying all night; answers rationally; complains of hunger and thirst; left orbicularis palpebrarum firmly contracted, drawing up the angles of the mouth, and giving an expression of a grin to the face; right pupil strongly contracted, left excessively dilated. No contraction or paralysis of limbs; right arm much more sensible than the left; picks his mouth and nose as before. Skin of body and forehead cool; no pulsation of arteries of head. Pulse very small and quick, 148. 150; respiration 36, without sighs; no delirium; much less stupid than yesterday. M. Guersent ordered the irrigation to be suspended in order to see if the child would sleep. Almond emulsion; decoction of marsh mallows; moderate diet.

Sept. 7th. Child looks much better; cried but little during the night; has slept a little; constantly carries both hands to summit and back of head, or picks the nose and lips; left cheek flushed, right pale; right pupil much more contracted than left; mouth deviates when he cries; no stiffness or paralysis, but slight trembling of arms on motion; no delirium, but does not answer as clearly as yesterday; skin a little warm; pulse 130, unequal in force; respiration deep, slightly irregular, 40; abdomen retracted; no stool; no vomiting. Decoct. mallows; emulsion 4 oz. Two injections containing sulphate quinine 8 grs.; extract of bark 8 grs.; resume the irrigations.

Sept. 8th. Slight convulsions yesterday evening; coma; pupils largely dilated; mouth spasmodically shut; face covered with abundant perspiration. The child has a slow automatic motion of the hands towards the face, and every now and then a convulsive shudder, the arms becoming stiff, the fingers strongly flexed, and the thumbs concealed beneath them; face and mouth occasionally drawn to the right side; skin warm; pulse imperceptible; respiration 42; no evacuations, but urinates freely; retraction of abdomen ceased.

Sept. 9th. Convulsions last night; insensibility perfect; left orbicularis muscle does not resist the finger; left pupil widely dilated and insensible; right orbicularis contracts; right pupil contracted and sensible; cornea dull, growing opaque; mouth and tongue excessively dry and brown; no stiffness; no paralysis; cutaneous sensibility preserved; pulse more distinct than yesterday, but excessively small and rapid; respiration regular, 34. Continue remedies.

Sept. 10th. The boy lies quietly in bed, with a constant trembling motion of the hands; no convulsions; arms occasionally perfectly stiff, and fingers flexed; deglutition of fluids; pupils as above; face pale; forehead very warm; right cheek warm and moist, left deadly cold; left arm warm and relaxed, right cold and stiff; no stiffness in lower extremities; no evacuation; pulse 150.156; respiration 36, pretty free. At 3 P. M. the face and chest were moist, forehead burning hot; variations in the temperature of the skin nearly as above, but more marked; respiration getting embarrassed; a fatal sign. Death at half past 6 P. M.

*Post mortem examination* 24 hours after death. *Head.* Membranes lining the upper surface of the brain dry and injected; convolutions flattened as if pressed against the cranium; two or three drachms of fluid in the occipital fossæ. Anterior and posterior lobes of cerebrum healthy; but near the fissure of Sylvius, the pia mater was thickened, firm, of a yellow opaque colour, and contained numerous miliary tubercles. Near the sides of the hemisphere this appearance was gradually lost, but the number of miliary tubercles was particularly great on the right side. The pia matter between the convolutions was deeply injected, but free from granulations. On the posterior edge of the left middle lobe there was a considerable exudation of sero-sanguineous fluid into the pia mater, and in one of the anfractuosities three tubercles the size of peas. The cortical substance of the brain was moist and not firm. The inferior part of both lobes was pale and diffluent; the central portions, corpus callosum, fornix, &c., were also quite soft; the walls of the ventricles were soft; they contained but a small quantity of fluid. Cerebellum healthy; but the pia mater which passed from the cerebrum to the cerebellum, near the crura of the latter, was infiltrated with the yellow matter before described. *Spinal marrow.* Membranes healthy; no granulations; upper portion firm; inferior much softer, but not injected.

Two small tubercles were found in the chest; one in the lungs, and one in a bronchial gland.

Mucous membrane of stomach perfectly healthy; that of small intestines injected in portions, softened throughout; a small ulceration was discovered at the commencement of the jejunum, and some of Peyer's glands were red and tumid, but none ulcerated. No tubercles in mesenteric glands, or in other parts of abdomen; all the other viscera healthy.—*Lon. Lancet.*

---

*Case of abscess in the sub-peritoneal cellular tissue, resembling Acute Peritonitis, cured by an opening at the umbilicus.* By M. Bricheteau.—Miss Henrietta D., æt. 17 years, of a scrofulous diathesis; her mother was rickety and scrofulous, and her father advanced in age; she also had suffered from dysmenorrhæa.

May 17, 1839. Seized with acute pain in the abdomen, which continued during the night, causing her to cry out, and preventing sleep. The following night she was in the same condition. Called to attend her on the 2d day, (20th.) I found the abdomen so tender that she could not bear the least pressure on any part of it; heat of skin trifling; pulse hardly accelerated; frequent bilious vomiting; great anxiety, without much change in the countenance.

Twenty leeches to the abdomen, 15 to the anus; hip bath; emollient injections and fomentations. Absolute diet; mucilaginous drinks.

May 21st. Ordered a few drops of laudanum of Rousseau, to quiet the vomiting and anxiety, from which she suffered much.

This treatment (omitting the leeches,) was continued for several days, and with apparent success. The pain in the abdomen and the vomiting ceased; pressure no longer caused pain. But the pulse remained accelerated 100 a minute; the respiration frequent, 44. The appetite returned, and the patient began to take soup after a week had passed.

But the pain in the abdomen soon returned, with swelling and tension; inability to sit up; vomiting; fever; pulse more frequent. The patient lay constantly on the right side; thirst; much fever; constantly complaining, without indicating the precise seat of her suffering; two or three alvine dejections daily. *Leeches* were again applied, particularly over the region of the spleen, where the pain and tension appeared to be most marked. Half-baths; fomentations; emollient injections; rigorous diet.

The pulse became more frequent, 120.140; varying, however, as also the pain in the abdomen, which would disappear in one spot and return in another; thirst always urgent; vomiting less frequent; sleep tolerable; ability to sit up a quarter of an hour each day; constipation in place of the slight diarrhœa; urine abundant, without particular character. As this new attack subsided, the disposition to vomit did not subside entirely; the tongue continued coated; the thirst considerable; the abdomen distended, but sonorous only on the left side. The patient lay constantly on the right side.

On the 18th day an attack of acute pleuritis supervened, which was treated with a blister and with calomel.

The patient continued to remain about in the same condition; pulse 120; skin rather hot; complexion clear; irregular accesses of fever and restlessness during the night; still some sleep. The abdomen constantly distended, and sonorous only on the left side; flatness on the right.

June 1st. M. Marjolin called in consultation; fluctuation; unfavourable prognosis; supposition of peritonitis with effusion. Ordered frictions with mercurial ointment and extract of belladonna; calomel in small doses.

June 12th. Skin of the umbilicus thinned and pointing. Size of abdomen somewhat less; tenderness diminished.

June 14th. An abscess discharged itself at the umbilicus; an enormous quantity of pus, estimated at several wash-hand-basins full, escaped, the patient became faint, but soon recovered, and fell quietly asleep. The matter discharged was healthy pus.

16th. M. Marjolin was again called in consultation. On pressing the abdomen a large quantity of healthy pus still gushed out. The patient had still nausea and vomiting, with partial cold sweats. She was ordered nourishing diet, quinine, &c,, and to lie upon her face, the abdomen being covered with a poultice.

26th. The patient has experienced a relapse; she has become restless, and complains of pain; she is delirious, and has been vomiting; the abscess had ceased to discharge.

29th. She is much improved, but the abscess has not again discharged; the urine has begun to deposit an abundant mucous sediment. Pulse 120.

July 8th. After some further variations in the condition of the patient, convalesence has now fairly commenced; pulse 96. In fact, the patient having spent the months of August and September in the country, has returned to Paris, perfectly recovered.—*Gaz. Med.*

*Post mortem Appearances found after Burns.*—Mr. Long, in a very interesting paper, read before the Liverpool Medical Association, has collected and compared a large number of post-mortem examinations, in which death was the result of burns. The following will be found to be a condensed summary of his conclusions on this subject.

He divides his cases into three classes.—1st. Those that died within 48 hours, (although this period is variable,) from the irritation of the cutaneous envelope and the re-percussion of the fluids to the interior. 2d. Those that died after reaction had taken place, with phenomena referable to the nervous system, or to the production of inflammation in those organs which are already the seat of congestion; these phenomena being ordinarily revealed about the fourth or fifth day. 3d. Those patients who survived the period of irritation and congestion, and that of reaction and inflammation, to fall victims to long protracted suppuration, with such lesions as are commonly found to exist in those who die of chronic diseases, especially profound alterations of the mucous coat of the small intestines. Finally, according to MM. Margolin and Olivier, patients may die after the wounds from extensive burns were nearly or entirely cicatrized. Delpech, in examining such cases, has discovered no organic lesion to account for death, but thinks it may be attributed to the disturbance of the functions of the skin.

I. Eleven post-mortem examinations of the first class, all presented marked congestion of internal organs. They all terminated fatally within 48 hours, and eight of them, which terminated within thirteen hours, presented more marked congestions, than the three which survived several hours longer. Seven of these were females, and only two males; in two, the sex was not specified; seven were under fifteen years of age; four above fifteen. In one, no examination of the head was made; while, in ten, lesions of various extent were found from simple engorgement of the sinuses to the effusion of bloody serum into the ventricles and at the base of the brain. In one, no examination of the chest was made; in one, no lesion was found; while in nine, lesions principally indicating congestion to various extent, existed. The same was true, precisely, with regard to the abdomen as to the chest.

II. Fifteen cases terminated fatally during the second period, viz. at variable periods, after 48 hours from the time of the accident. Of these, three died during the period of reaction, and twelve during that of inflammation. In seven, the internal lesion corresponded more or less in locality, to the external injury. Twelve of the cases were females, and two males; in one, the sex is not specified. Seven were under fifteen years of age, and eight above that age. In six of these cases, lesions of the brain were found; in three, this organ was not examined. In not more than one of these cases did distinct inflammation of the brain (arachnitis) exist, the remainder exhibiting instances of congestion more or less marked. In seven cases, lesions of the organ of the chest were found; in three, this cavity was not examined. These lesions were of a much more marked inflammatory kind than those of the brain; in five cases, acute inflammation of the lungs or pleura existed. In ten cases, lesions of the organs contained in the abdomen were found to exist; no mention of any lesion in one; nothing found in four; so that probably these organs were examined with more or less care in every case. In these cases the mucous membrane of the stomach was inflamed four times, and in one perforated; mucous membrane of the intestines inflamed seven times; in two, it was gangrenous; in two the duodenum was perforated; in four cases acute peritonitis existed.

III. But one of the cases terminated during the period of exhaustion; a child æt. 8 years, who died on the 35th day. In this case, recent inflammation of the pleura was the only lesion noticed.

From the above statements, the following conclusions may be derived:—1st. That more females are burned than males, and that of those burned, a greater proportion of females die than males. This is easily explained; the occupation of females exposing them more to fire, and the nature of their dress rendering their burns more severe. 2d. That death may take place during the period of either congestion and irritation, reaction and inflammation, or exhaustion. 3d. That the greatest number, nearly half, die during the period of congestion; nearly one-third during the period of inflammation; rather more than one-fifth during the period of reaction; and very few during the period of exhaustion. 4th. That more individuals above the age of fifteen, than under that age, die during the period of inflammation; more die under that age than above it, during the period of reaction; and that during the period of congestion, the proportion of deaths above or below fifteen years, offers but a slight difference, there being fourteen cases above fifteen years, and eleven cases under that age. 5th. That in almost every burn, indeed in every burn, lesions of one or more of the viscera contained in the three great cavities exist, being, according to their frequency, as follows: abdomen, chest, head. 6th. That the lesions of the different tissues contained in the abdomen, are in the following order: mucous membranes, serous membranes, parenchymatous tissues; in the chest it is quite the reverse, viz. parenchymatous tissues, serous tissues, and lastly mucous; in the head—membranes, brain. 7th. That the seat of internal inflammation corresponds sufficiently often with the external position of the burn, but that in a precisely equal number of instances no such correspondence can be traced.—*Med. Gaz.*

*Case of Abscess of the Pharynx.* By C. FLEMMING, M. D.—The premonitory symptoms of this disease, according to Dr. F., are, local uneasiness, which is common to all affections of the throat, complained of, or otherwise, according to the age of the child, and on examination not accompanied by proportionate visible lesion. The *essential* symptoms often supervene very suddenly, indicated by derangement of the cerebral, circulating, and respiratory systems, alternating with a comparatively healthy condition of those systems, according to the alteration in the position of the individual. Fixed and retracted state of the head; with rigidity of the muscles at the back of the neck, and more or less locked state of the jaws. Painful deglutition; impossibility of swallowing solids, and fluids convulsively darted forward through the nose and mouth. Repeated acts of deglutition without the presence of any fluid in the mouth, and on examination of the fauces, a firm projecting tumour felt beyond the base of the tongue, and if seen, presenting a smooth, rounded, highly vascular appearance behind the soft palate; usually occupying the median line, but occasionally inclining to either side. These essential symptoms accompanied with the ordinary characteristics of suppurative fever.

The following cases illustrate this disease:

The first case was a boy. His age was three years and a half, and in appearance he was healthy. The premonitory symptoms of his attack, at first mild, after about thirty-six hours, assumed most intense severity, and without unnecessarily particularizing their progress, it may be stated, that the most aggravated form of high inflammatory fever set in, principally engaging the cerebral organs, and requiring the most energetic treatment to combat it. On about the fourth day, convalescence appeared established, and Dr. Crampton (whose valuable assistance I had throughout the progress of this case) discontinued his daily attendance.

From day to day a peculiar fixed position of the head, and stiffness in the neck, now attracted attention. The head was drawn back. The muscles, at first tense, became completely and permanently rigid, and the movements of the head painful, and remarkably limited. Soreness in the throat was complained of, and also great difficulty in swallowing, at times accompanied with violent spasmodic efforts. There was no cough, and the voice remained perfect. The articulation became remarkable,—the words being as if drawled out with pain and difficulty, and at times perfectly unintelligible.

Repeated and careful examination of the fauces and neck, could not detect any apparent local cause for those symptoms, which, with varied degrees of intensity, advanced, producing equally alarming constitutional disturbance and debility.

At first, disposed to attribute them to concurrent local causes, such as the quantity of mercury administered during the acute illness of the child, the cold from the renewed application of ice to the head, or some partial internal effusion, the result of the acute inflammatory attack, more serious mischief was now apprehended from their increasing severity and permanency. The treatment adopted was principally with the view of promoting the absorption of any fluid effused, and consisted chiefly in the exhibition of mild mercurial alteratives, and the application of counter-irritants to the region of the occiput.

On about the tenth day, the symptoms had reached their acmé; the child, emaciated and weakened, had no relish for food, and appeared to drink merely to allay thirst, the efforts at swallowing being convulsive and painful. He was now in a perfect state of somnolency, regardless of every thing about him, when accidentally, whilst sitting beside his bed, I perceived, that *position* most remarkably influenced the severity of the prominent symptoms. Stupor in the recumbent posture, almost amounting to perfect coma, in the sitting, or even semi-erect, resolved itself into a comparative sensibility. Respiration slow, laboured, and stertorous, or rather roaring, (as described by the attendants on the child,) in the former position, became comparatively tranquil in the latter, and a pulse in the one, ranging only a beat or so above forty, in the other, assumed a more natural character. Again, fluids were more frequently darted convulsively forwards through the nostrils or mouth, than passed into the stomach, or were ejected, as in the act of vomiting, and the recurrence of the symptoms of cerebral compression took place on returning to the recumbent posture, which, for the last three days, had been almost the permanent one.

I now considered that this relation of symptoms might still be caused by mechanical obstruction in the pharynx, although repeated examinations on former occasions did not lead me to this conclusion. An additional obstacle presented itself in the fixed position of the jaws, so that it was only by considerable force I could so far separate them as to admit of even getting my little finger between them. On forcing it back, I accidentally, but distinctly, felt a tumefaction beyond the base of the tongue, giving, as well as a compressed finger could indicate it, a sense of yielding. To get a view of it, was utterly impossible. The soft palate and uvula were easily discernible, but the depression of the tongue gave so much pain, and the separation of the jaws was so very limited, that further investigation was totally out of the question. Indeed, in addition, the evidence, even from touch, was necessarily momentary, from the severe paroxysms of dyspnœa attendant on the examination.

Although I had never heard of, nor witnessed a case of the kind before, in children, it at once occurred to me that this might be an abscess at the back of the pharynx, mechanically producing the above symptoms, and having stated this as my opinion to the family, the assistance of Dr. Crampton and Mr. Cusack was immediately procured. After a patient, though extremely unsatisfactory examination, they coincided in opinion with me as to the presence of a tumour in the situation alluded to, and it was determined that I should perforate it with an explorator which I had provided for the purpose, with the view of ascertaining its actual nature; a doubt existing on this head, not alone from the extreme firmness of the tumour communicating a very indistinct sense of fluctuation, but also on account of its probable anomalous nature from the previous acute and present chronic cephalic symptoms. With every necessary precaution I accomplished this object, though with considerable difficulty, and to my great gratification, witnessed the sudden gushing forth of a large quantity of healthy purulent matter. The whole features of the case were almost instantaneously altered. The somnolency was removed, deglutition was facilitated, and more cheering prospects manifested themselves. Nourishment was freely given throughout the day, and quinine administered in small and repeated doses.

At my evening visit I perceived that the stertorous breathing had returned, and that the more prominent symptoms which had ceased since the operation, were again in some degree present. I examined the throat, and fortunately found the separation of the jaws now accomplished with ease. The abscess was again filled, with the opening closed. I introduced a carefully protected sharp-pointed bistoury into the site of the opening, and freely enlarged it downwards. The relief was instantaneous. I now directed the trunk of the child to be elevated as much as possible, and the head depressed. The night was passed comparatively tranquil; the quantity of matter which escaped through the mouth was considerable, largely staining the pillow. The next day the boy was able to play with his brothers, and subsequently his improvement was progressive, though slow.

He is now a fine healthy boy. I do not particularize the treatment adopted during his convalescence; there was nothing peculiar in it, its principal object being to improve the general health.

The next case which I shall select, is that of a boy aged seven months, proving the remarkable fact of the occurrence of such an affection during the first period of childhood, as the former does, during the second.

In April, 1838, I was sent for to see this child by the father, who stated that he had great apprehension his little boy was labouring under water on the brain: that many children of his immediate family had fallen victims to it, and that the symptoms under which this child laboured, were exactly those by which the attacks of the former had been ushered in. On visiting the child, I found every indication of gastro-enteric derangement, so common at this period of life, and very suspicious cerebral complication, rendered more so from the fact of hereditary pre-disposition. In addition I found, that some lymphatic glands, on the left side of the neck near the angle of the jaw, were enlarged and painful, evidently depending on ulceration behind the corresponding ear. The mouth, fauces, and pharynx, were free from lesion, and one of the incisors on the lower jaw had just made its appearance.

The treatment was principally directed to the abdominal system, and to the relief of the glandular irritation noted. After a few days, improvement was so manifest, that I had omitted a visit on Friday.

On Saturday morning I received a hurried message to see the child, and found that the more alarming symptoms had all returned during the previous night, that the restlessness was incessant,—that the vomiting was constant,—that the flushing of the face was renewed,—that the breathing was loud, laboured, and very irregular during the night,—and that he constantly started from most disturbed sleep, which would only be tolerated in the nurse's arms; that every attempt at putting him in the cradle aggravated the pulmonary symptoms. In addition, I observed that the head of the child was rather drawn back, and that the chin projected somewhat unnaturally. He immediately screamed when the jaws were attempted to be separated, and in the region of the neck there was the greatest tenderness, particularly over the glands above alluded to. The integuments were free from discoloration, yet still the tumefaction was decidedly increased, and the slightest motion of the head appeared to give great pain.

At the moment, I was disposed to attribute the recurrence of those symptoms to a smart attack of inflammation in these glands, and was led to hope that the combatting it would relieve them. The treatment was accordingly directed with that object in view. Leeches were applied; fomentations and poultices used, and a smart mercurial purgative administered.

*Sunday.*—Night spent wretchedly; no alleviation of symptoms, with the exception of those connected with the inflamed glands; they are better: the other symptoms are, if possible, more aggravated. In addition to those enumerated in the report of yesterday, there is now a gurgling noise in the fauces, as if from accumulated mucus, and throughout the lungs there is evidence of considerable effusion into the larger bronchial tubes; there are repeated and apparently painful and difficult efforts at swallowing, accompanied with frightful paroxysms of dyspnœa occurring at irregular intervals, during which the countenance becomes suffused, purple, and almost convulsed, and it is remarked that those immediately supervene on attempting to place the child in the cradle; there s incapability of sucking, though great desire for the breast, the nipple of which is seized with avidity, and equally rapidly ejected with a sudden and spasmodic regurgitation of the milk; any fluid placed in the mouth, either remains for a short time, and then gradually dribbles out, or otherwise produces a paroxysm accompanied with similar phenomena. At the moment of my visit, the repeated exertions of the child at the attempt of swallowing, the severe dyspnœa, and the great accumulation of mucus in the fauces, with the very restless state of the child, led me to apprehend the supervention of a fit of convulsions. I thought I recognised some of the features of the above case, when, from some unintentional act in my examination, a most severe paroxysm supervened. The child appeared suffocating: I rapidly passed my finger into the fauces, and feeling a fulness, I made pressure against it, which was increased by a convulsive effort of the child; a sudden discharge of purulent matter got exit through the nostrils, and temporary relief was obtained, until I procured the additional assistance of Sir Henry Marsh and Mr. Cusack.

Perhaps about an hour or so had elapsed from the above occurrence, when we met in consultation. At this time the breathing, though principally nasal, was more tranquil; and a small quantity of fluid had been swallowed, but with much difficulty. The appearance of the child could not but make an impression upon those who saw him. The nostrils were filled with matter which trickled down the lip; any attempt to place him in a recumbent posture was instantly followed by frightful dyspnœa, rendered still more serious from the great accumulation of mucus in the fauces. I directed attention to the throat, but notwithstanding every effort, no accurate view could be had of the back of the pharynx. The narrow space behind the root of the tongue was filled with pus and bubbles of frothy tenacious saliva, to clear which away repeated unsuccessful attempts were made. Here the freedom of separation of the jaws allowed of free, though rapid examination of the fauces, but the back of the pharynx could not be seen. I, however, felt a distinct tumefaction, and failing to puncture it with the grooved curette, as in the former case, I was obliged to rest satisfied with what had been done, arranging to watch the progress of the symptoms, and to support the child by every possible means, by introducing fluids through a tube passed through the nares, and by broth enemata; to be prepared, if necessary, to open the trachea, should any fresh symptoms of suffocation supervene; and in addition, to keep constantly cleared away the accumulating phlegm at the back of the throat.

By visiting at short intervals, and carefully enforcing the above injunctions, the strength was supported, and the symptoms to a certain extent stayed. Next day they were stationary, though it was quite evident that considerable obstruction yet existed in the throat; however, the strength was improved, and the countenance of the child decidedly better. Another day passed without any material change, when the discharge from the nostrils ceased, and evidently, any opening made, or rather the ruptured portion of the sac, had closed. Difficult respiration in any but the erect posture, or on an inclined plane with the head considerably depressed, recurred. Perfect inability of sucking and swallowing again set in, and suffocation appeared impending when Mr. Cusack saw the child, and was still more satisfied of the presence of a tumour at the back of the pharynx. It was so tense and so unyielding, that, did not the history of the case justify the presumption that matter was present, the absence of any sense of fluctuation would have caused extreme doubt; another difficulty presented itself in its being below the level of the tongue. The very limited space to operate in, together with the risk of wounding the neighbouring vessels, on account of the disposition of the swelling rather from the meridian line towards the left side, suggested the propriety of selecting some instrument, the action of which could be accurately gauged. That which I had used in the former case was objectionable not alone from the want of sufficient command of it from its conformation, but also from its shape. It was agreed that delay might be safely hazarded until next day, leaving word, however, that should any urgent symptom set in, I should be informed.

Next day, I found that throughout the night great apprehensions were entertained lest suffocation should have taken place. All other bad symptoms remained, if not aggravated, at least stationary; and having arranged in the interim with Mr. Cusack, an instrument was contrived which succeeded most admirably. It consisted of a trochar about four inches long, one extremity of the canula being slightly curved, the other with a ring on its upper surface to receive the fore-finger; into this canula was passed a jointed stilette, with, at its opposite extremity, a ring for the thumb, and a movable screw to graduate the projection of its point. Mr. Cusack having firmly supported the head of the child, I passed the fore-finger of the left hand towards the back of the pharynx, there resting the point of it, and guiding the armed trochar, with the concealed stilette, along it, accurately fixed it on the tumour, pressed forward the stilette to its limited mark, and withdrawing it by an opposite manœuvre, was gratified to see *a quantity of healthy purulent matter darted forwards on the child's clothes.*

The relief was immediate; the hæmorrhage trifling; and the result permanently successful. In this case it was unnecessary to renew the opening; the discharge, at first temporarily ceasing, returned, and the cure was rapid.

The boy is now a fine healthy boy. The constitutional treatment was similar to that adopted in the last case.

Such is the history of two extreme cases of *acute* abscesses at the back of the pharynx, occurring in children, selected from others of the same nature, which I have witnessed within the last three years, and necessarily with opportunities comparatively limited. I have brought them forward as remarkably illustrative of the symptoms attendant on their progress; as novel at that period of life, in the records of medicine, as far as I have been enabled to learn from the investigations I have made, and as corroborated by the testimony of others.—*Dub. Jour.*

---

*On the Bilious Remittent Fevers of Peru.* By ARCHIBALD SMITH, M. D.—The bilious remittent fevers prevail most in Lima and on the coast generally, during the sultry months of January, February, and the earlier weeks of March, when they are very formidable, though some severe examples of this kind are also observed to occur about the time of the vernal and autumnal equinoxes, when intermittents are abroad in every street, and often put on, as already observed, the disguise of a continued or remittent fever during the first few days of their attack.

In progress of treatment and cure even the more intense remittents, with decided bilious character, which prevail during the warmer months, frequently dwindle into a tame quotidian or tertian form: but in general their features are sufficiently peculiar and well-marked, and they deserve separate consideration.

I shall relate a few cases by means of which accurate notions of these fevers and their common transitions of type may be conveyed to the reader; and to give a fair account of such cases I rather choose to be somewhat minute than to pass over details with great brevity.

*Case* 1.—A young man, native of Lima, and engaged in commercial pursuits, had occasion in spring, (in the month of October,) to ride to Callao, and there he dined on beans and salad, and immediately after drank a few drams of the spirit of the country, called Aguardiente, which was medicated with anise-seed. After all this, in course of the day, he rode back to the city, and at night he felt feverish. Next morning I was called to visit him, and on my arrival the fever had remitted, but the patient and his attendants agreed in stating that it had been very sharp over night. On examination it appeared that the colon was greatly inflated, forming a large sensitive tumour, where it traverses the right hypochondriac region. His tongue was foul, and of a yellowish appearance; his alvine motions were frequent and bilious, and he had occasional vomiting of a bitter or bilious fluid. Caldo, or mutton-tea, his stomach rejected, and it was only such farinaceous articles as rice pap or arrow-root that would rest upon it. Several emollient enemata were administered by the female attendants.

Having found him as described, I took advantage of the existing remission, and prescribed, for immediate use, a bolus of conserve of roses, with eight grains of calomel, as an appropriate purgative in the irritable state of the patient's stomach.

This evacuant carried away copious bilious stools, and produced great relief; for vomiting entirely ceased, and the stomach became so far quieted as to bear *caldo* without nausea or inconvenience; the distended colon sunk to its usual level, and no longer simulated an enlarged liver, as it had done on the preceding day; perspiration flowed easily, which it did not till the bowels were acted upon by the calomel; and the result of all these changes was that the fever quickly assumed the intermittent form. After this transformation the accessions continued to be nocturnal; but these were mild, short, and few in number: they were ushered in by a pain that darted along the right shoulder.

The young gentleman was kept for a few days on spare diet; and was allowed lemonade or tamarind-water for common drink. The fever was soon removed by the infusion of bark, to which was added as much sulphate of potash as served to regulate the daily action of the bowels.

*Case* 2.—A Serrano or mountaineer trader came, in the month of February, to one of the taverns or *Tambos* in Lima, where muleteers and traders from inland districts usually take up their quarters.

For the first four days after his arrival in town, he kept beating about the streets, and making such purchases as suited his purpose, in course of which he was necessarily exposed to the sun, strongly reflected from the walls and pavement. During these busy days he experienced a good deal of thirst; and while his *peones* or servants indulged in succulent fruits, he himself eat daily one of the very large melons, so common in the capital at this season of the year, as a cooling accompaniment to a fair allowance of cheese and other dry food, as "cancha and charqui,"* &c., to which men of his mode of life are much used among the hills.

On the fifth day of this sort of living and exertion, he was seized with strong flushing and slight chills, which lasted all the first day of his illness; while, for several days after, he always felt great oppression, weight, and tightness about the epigastrium and belly generally. At the same time his tongue was furred and yellowish; nausea frequent; thirst excessive, and call for cold water urgent; but his nurse was afraid to let him have it; his left eye was blood-shot, and the whole left side of the head

* "Cancha" means toasted maize, and "charqui" sun-dried meat. Charqui is corrupted into *jerked* beef by the English seamen.

ached severely; his skin continued to be, as he expressed it, burning hot for several days; the urine was very high coloured; belly bound, and several laxative or emollient enemata were administered at the suggestion of the female attendant, without adequate relief. Each successive night his agitation, anxiety, and fever rose to a degree that approached to delirium, and his extreme restlessness continued till the coming on of dawn, when, without having sweated, though the skin became somewhat humid, the violence of the fever declined.

Things were stated to have remained so for three days before I saw the patient, and at my first visit I found him with a strong accelerated pulse, and very considerable fever; yet, judging from the narrative of his case, his fever was at this time in a state of comparative calmness or remission, and the evening exacerbation was expected to recur in the course of three or four hours after. No time was to be lost in the treatment of the case. Enemata had already proved unavailing; and therefore the bowels, in which the disease appeared to have originated, required to be unloaded by some other means without delay. The lancet should here have taken precedence of the purgative; but there was an obstacle, which will immediately suggest itself to the native practitioner, viz. the *empacho* or surfeit of melon and cheese, &c., for it is a rule among the vulgar never to consent to be bled when their bowels are loaded, or supposed to be so with feculent accumulations; and hence this patient was not bled.

A dose of compound powder of jalap, mixed in tamarind and mallow-water, was given, and in less than two hours it procured a free discharge of five abundant and thin, but feculent motions of a yellow colour; and, being then allowed a basin of weak chicken soup, he voided, in course of the evening, eight or nine more stools, which had precisely the appearance of dark-jaundiced urine, without any trace or admixture of mucous or feculent matter interspersed. The patient, after this copious discharge from the bowels, perspired profusely all night, and next morning he had neither fever nor hemicrania, but the tongue remained yellow and furred. Since the free perspiration commenced, there were no more stools, and conceiving it probable that the fever would not return, the patient was only directed to alternate linseed-water with a thin and watery chicken-soup, or "*caldo de gallina*," in the usual manner. But, on the day following, when the patient was again visited, he related:—"Yesterday, at 4 in the afternoon, I was overtaken, when I little expected it, by feverish heat, much thirst, and an inward heat of the belly, which was removed by the interference of my kind nurse, who anointed the whole abdomen with rose-ointment and almond oil. After this, I felt much wind in the bowels; and of this sort of disturbance I was also relieved by an enema of alorba (*Fœnum græcum*) linseed, and sugar, with the addition of a little rose-oil, but without voiding by stool any thing to signify. I perspired in the early part of the night, but not freely, nor so as to refresh or cool me. At midnight, the fever ascended to a high pitch. The upper half of my body perspired somewhat, but the lower half perspired none; and in the feet there was a burning heat. One eye and half of my head were affected with the most acute pain; a perfect desperation seized me; my agitation became intolerable; my frame was thrown into involuntary jerks; my mind was full of illusions; and my apartment seemed to be filled with armed soldiers, and other equally visionary objects. I had nausea and a slight cough. At length a general sweat broke out, and then I fell into an unsettled slumber till day-light, when I felt refreshed, but not so cool as I did yesterday when you saw me."

Having thus finished his narrative I found that he had still some relics of fever, and more or less hemicrania. I prescribed the infusion of bark, with a little sulphate of magnesia and sulphate of quinine, to which a few drops of dilute sulphuric acid were added; and this he was to take at proper intervals, when free of any considerable fever. Shortly after this receipt was left, the paroxysm anticipated its usual hour of approach, and rose as on preceding days without any notable cold stage. The nurse in attendance, finding his skin hot and mouth dry, gave the patient, as instructed to do in the event of the accession returning, iced-water to drink; and he being a mountaineer, not accustomed to the daily use of ice like the people of the coast, this frigid draught, almost instantly it was taken, determined a very strong cold fit, which lasted nearly fifteen minutes, and this was followed by a smart hot fit, but it did not last long. The sweat that now ensued was free and refreshing, and it gradually lapsed into a gentle diaphoresis, and then the patient fell asleep, and slept all night. In the morning he awoke without complaint, and took the above mixture of salts and bark, &c., in doses of a wine glass full, repeated every two hours. He had no further return of fever or any bad result from it, but immediately resumed his commercial occupations.

I would here remark that the high degree of irritation exhibited in the above case would have deterred the followers of Broussais in Peru from using a purgative at all, or, at least, until preceded by blood-letting. But I have had frequent opportunity of observing that this sort of general febrile irritation, kept up in a great measure by a disordered state of the *primæ viæ*, unconnected with local inflammation, may be mitigated at once by an efficient purgative, though unaided by bleeding, as in the above instance.

*Case* 3.—The following account was given by the young woman in attendance on the sick, who had the appearance of being a respectable tradesman in the prime of life. The case occurred in February.

The patient had an *empacho*, which evinced itself by great loathing of food, white tongue, bitter taste in the mouth, uneasiness at the pit of the stomach, and a confined state of the bowels, which were filled with wind,—'*aventada.*' I let him have some ordinary clysters, by aid of which he evacuated much: the first stools being hard and scybalous, and those that followed loose and yellow. After this, he felt in a few days as if well; and therefore he ventured to wash his hands and face in cold water, and in the evening he stepped into the court yard in shirt sleeves. On this very evening sprung up a fever, which must have proceeded from his having washed himself in cold water.

Five days, with to-day, these continuous fevers, *las fievres*, have lasted. The one follows the other so closely that he is never free from them; but at one or two o'clock in the day they begin to rise, and continue increasing until midnight, occasioning great inquietude and extreme impatience, amounting to desperation.

His skin is burning; sometimes he complains of his head, and at other times he does not; but his sleep has utterly abandoned him. During the increment of fever he has an inclination to retch. At day-break, a slight exudation somewhat softens the skin, (only one morning has he been so fortunate as to sweat more freely, when he had to change two shirts,) and then it is that he falls over into a dozy state, and that the fever subsides considerabiy without ever leaving him altogether.

At the same time when the fever is rising to its highest degree, he feels great heat inwardly, and expresses an eager desire to be allowed to drink iced-water, but I only give him plain cold water with a bit of sugar.—"No le doy mas que un teroncito de azucar con agua para chupar." His urine is fiery and highly coloured, *incendida*; and as often as he takes any simple drink, or panada, which constitutes his food, he voids a watery yellow-coloured stool.

I visited this patient about five in the afternoon, when I received the above account of his case, and was told that the exacerbation had just commenced. His pulse was at this time one hundred and twelve, somewhat hard and contracted; tongue nearly clean and humid; he implored to have fresh air admitted into his apartment, but his attendant was afraid to admit air from a high window in the room, lest it might induce *ayre* in the sick—that is a sort of palsy occasionally produced by exposing the warm body to a current of cool air.

In the lower belly he felt pain on pressure, which did not exist from the beginning, and, fearing the existence of inflammation there, cold iced-drinks were prohibited. I suspected that the incessant use of enemata might have had something to do with the tenderness now experienced in the hypogastrium, and therefore prohibited them, and recommended that he should be bled without delay.

I may notice it as an instance of popular prejudice, that next day when I returned to visit this person, I could not see him. I was informed by the young woman who waited upon him, that, during night, when the fever had attained its acmé, he became quite insensible, and that his mother (who probably considered his case to be a bad *empacho*) would not consent to have him bled, because, she said, she was under no necessity "*no tenia necesidad*" to kill her son, by bleeding him at the bidding of any one; and therefore she called in another doctor.

I had the curiosity afterwards to inquire concerning the termination of the above case, in which the appearance of the stools particularly indicated a bilious affection, and learned that, though not bled, his disease had ultimetely subsided into an intermittent, when it was cured by some preparation of bark.

*Case* 4.—In consultation, I had to deliberate on the case of a young and robust European, for some years settled in Lima, while labouring under one of the very vehement bilious remittents of the country with which he was attacked in the spring.

Before I was called to join in consultation, this gentleman had been many days excessively ill, and treated by several physicians; but the case would not yield to general bleeding, warm baths, ptisans, and refrigerants, clysters, oily embrocations, cataplasms, and sinapisms, &c., which had all been tried. When I first saw him, the fever was lively and the remission ill defined; the sensibility of the liver was much excited, and the stomach rejected every thing; bile was vomited by cupfuls; the ear was offended by the softest sounds, those of an affectionate voice; the eye could not tolerate a ray of light; the head ached until it obliged the patient to ejaculate in agony, and the very integuments over the forehead were tumid and greatly swelled.

The *junta* were in doubt about the propriety of further general bleeding. Local bleeding was proposed, but rejected by a majority of votes. The consultation was broken up, and the friends of the sick intrusted me with the charge of the patient. Being thus at liberty to act independently, cupping glasses were promptly applied to the nape of the neck, and blood was abstracted pretty largely, which produced the most striking effects. The swelled integuments of the forehead, from being tense and prominent, became, before the cupping was concluded, soft and yielding; the senses of hearing and seeing quickly lost their extreme morbid acuteness; the headach became moderated; and the stomach was so far composed as to bear a cupful of fresh tepid whey three or four times a day, and in each cupful was dropped three drops of Fowler's arsenical solution.

By these means, the imperfect remissions in course of some days became distinct intermissions; and after a short time, with little more aid than an emollient application over the seat of the stomach and mild opening clysters, the fever disappeared, and the patient went for change of air to a town in the interior, called Canta, where he soon recruited strength, and after a few weeks' absence he returned to Lima.

The above cases may serve to give an idea of the variety and intensity under which the bilious remittents appear in Lima, and they also illustrate the downward tendency which these fevers have towards the intermittent character, after the excitement of the system has been lowered by the help of remedies.

On various occasions, however, sufficiently urgent cases of remitting fever present themselves, and with no small disturbance of the cerebral functions, which I have seen suddenly relieved by the vapour bath, or gradually removed by the warm bath, depletion, and evaporating lotions, together with aperient, effervescing, and cooling drinks, without ever changing to the intermittent, or degenerating into the low continuous form, of which I shall soon take particular notice.

It may be worth while to observe in this place, that tartar emetic, in every form of combination, is almost prohibited in the fevers of Lima by experienced native practitioners. This remedy, whether in vinous or aqueous solution, is never considered eligible in fevers of a bilious character, as it is apt to increase the gastric disquiet, or to excite vomiting, which might prove very injurious when the sensibility of the brain is observed to be much augmented, as it usually is in bilious remittents. This practical consideration appears to have prejudiced native physicians against the use of James's powder, which is an antimonial that admits of very general and useful application in fevers, as it both opens the bowels and invites perspiration.

As regards the use of tartar emetic, however, the writer's experience only leads him to consider it inapplicable on the coast of Peru, in fevers attended with much gastric disturbance, and especially when the brain appears to be much affected, as already noticed, in fevers of a well marked bilious character.—*Edinburgh Med. and Surg. Journal.*

---

*On the Gastro-Enteric Fever of Peru.* By A. Smith, M. D.—As a consequence of an *empacho* from figs or other fruit eaten to excess, or received into a weakly digestive apparatus, we hear the natives complain of a fever which they vulgarly refer to the stomach and intestines together, under the name of *Fievre gastrica entripada*, of which the more scientific term, gastro-enteritis, is an exact translation. This fever appears to be only a more intense degree of what I have described as the simple gastric. It is commonly attended with evacuations, and after the fever has disappeared, I have been consulted regarding the evils it left behind; such as a sense of pain or uneasiness in the transverse arch of the colon; frequent attacks of indigestion, or irregular action of the bowels, being at one time bound, at other times too relaxed. And it is to be noticed that all this inconvenience may be suffered in defiance of the utmost attention to regimen, and no deviation on the part of the patient from the established dietetic rules laid down by the physician.

With regard to treatment, it is commonly confined to bleeding, warm baths, diaphoretic diluents, laxative enemata, cataplasms to the region of the stomach, and a spare farinaceous diet, with occasional use of purgatives. Most medical practitioners in Lima are agreed, and the author on practical grounds concurs in the opinion, that in the gastro-enteric fever with which they are familiar, the moderate use of mild purgatives, sufficient to carry off any undue fæcal accumulation or vitiated secretions in the intestinal passages, is an essential point of practice which can rarely be overlooked without detriment to the patient.

Some practitioners trust almost exclusively to the use of gum-water, and cupping or leeching the abdomen with cataplasms. But leeches cannot always be procured in Lima; and when they are to be found, they are often sickly, and always very high priced, because the mortality among the leeches from Europe is so great as to render it necessary to put a high price on those that survive. Leeches from Chili are of very inferior quality, and do not answer well in Peru.—*Ibid.*

---

*On the Gastro-Catarrhal Fever of Peru.* By Archibald Smith, M. D.—It often happens that cold is caught when the stomach is in a state of repletion; and soon after the individual affected may be seized with intense fever, attended with cough or catarrhal symptoms, complicated with tenderness in the epigastrium, or a marked derangement in the functions of the stomach or liver, &c. Such complications may be expressed under the general term gastro-catarrhal fever; but it is to be observed, that the fever so called may either be radically intermittent, or remittent in its nature, and assume either of these forms; or pass off by insensible gradations, like cases of common continued or sympathetic fever,—as local irritations or confirmed phlegmasia, accompanying the fever, are seen to subside under proper treatment.

Fevers of this complex character, like more simple *tercianas*, arise from a variety of external causes, such as enjoying in the warmth of summer the cool of the evening breeze and night air, in open balconies and ample corridors; changing winter to summer clothes too soon; sudden transitions in the air, marked by a day of sunny brightness, followed by others of a damp and cloudy atmosphere; cold bathing begun too early in the season, or remaining too long in the water at any season; and, lastly, from drinking cold water when overheated by exercise. Children, who are commonly eating at irregular hours, and pampered with sweetmeats, nuts, and fruit, &c., are exceedingly fond in days of sunshine, about the end of October, or early in November, of playing about the doors and court-yards till overheated, and then they run, as indeed they are apt to do at all seasons, into the canals in the streets, dashing on themselves cold water. The immediate result is, a constriction of the cutaneous vessels, or what the natives call stoppage of the pores, *tupimiento de poros*, or simply "Resfrio," which brings on a fever more or less severe, and commonly of a continued or slightly remitting type.

Affections of the kind called "*resfrio sobre el empacho*," or checked perspiration on a full stomach or loaded bowels, are most generally removed in a very easy and brief manner; namely, by warmth and rest in bed, and purgative clysters, followed by simple diluents, as linseed or mallow-water. I may add, that in such cases—when not accompanied with much gastric disorder—a few drops of antimonial or ipecacuan wine is an excellent adjunct to these diluents, intended to remove the constriction of the cutaneous exhalents and promote sweat,—an object which is further favoured, when necessary, by the foot-bath, or general warm bath, and a suitable purgative to clear out the bowels.

To checked perspiration during a full state of stomach and bowels, we may trace, after the Carnival sports, and on other occasions, very formidable cases of the gastro-catarrhal fever,—as I mean to illustrate more particularly. But this complication of fever, with local irritation or inflammation, which simultaneously affect the organs of respiration and digestion, may originate from several exciting or occasional causes such as I have already enumerated, without any evidence of a co-existing indigestion, or any undue repletion or accumulation of feculent matter in the bowels. It is, however, of practical moment to recollect, that in by far the majority of cases of the gastro-catarrhal fever, the bowels are overloaded, and require very early and special attention from the practitioner.

I may now offer a few practical examples of this disorder.

*Case* 1.—A portly woman, about 30 years of age, and of a light Zamba caste, made the following relation of her case in January, 1836:

"Three weeks ago I had bitter cause of disgust and disagreement with my husband, who keeps a shop in the Calle de ———. He took me by the hand, and conducted me to the street door, saying, 'Anda vete! begone from my sight! I like to live at my pleasure.'

"I repaired to the convent of ———, and while in this asylum, I one day bathed my feet in cold water, and combed my hair with the help of cold water to smooth it down.

"While thus engaged, the bells of the city rung '*Campanadas al fuego*,' or the fire-alarm; when, believing the enemy at our gates, I ascended to the roof of the convent, where I caught additional cold; for you will observe, that before this I was colded, '*resfriado*'—and, therefore, more liable to become subject to some serious ailment; especially at a time when my blood was boiling with indignation against a husband, who, at the close of fifteen years that we had lived together, ended our union by throwing me out to the street, 'porque no queria aguantar y ser su alcahueta,' because I would not brook to sanction his irregular amours.

"From all this, you see, it happened that my cough became very severe, and I was further affected by vomiting, purging, cramps in the legs, and my lips became livid; then arose a fever that burned me, and I was attacked by jaundice. At present I sweat continually and excessively, which was not the case at the commencement. The jaundice is retiring, the evacuations have nearly disappeared, and the cramps have entirely left me. But I have still the cough and vomiting, great disquietude, and want of sleep."

Here ended her narrative. But on patient inquiry and examination, I further ascertained that what she now ejected by vomiting was sometimes greenish, with a dark sediment, that looked like toasted meal; and at other times watery-like, with slimy or mucous matter floating in it. Her stools were loose and perfectly yellow; she felt no pain at the epigastrium, nor any where in the abdomen, though gently pressed upon. Her eyes were very yellow, and her expression of countenance anxious and unsettled; the tongue was yellow and furred; breathing oppressed when speaking; urine high coloured, and reported to have been only a few days before so jaundiced as to have tinged linen dipped in it of a deep yellow colour. Her pulse was strong and frequent, notwithstanding that sweat in large drops oozed at every pore as fast as she could wipe it away with her handkerchief. I observed her continually shifting posture, and she said that she tossed and turned all night. Whatever nourishment she attempted to take, as chicken-soup, panada, or rice pap, &c., her stomach rejected; but she had thirst, and wished for nothing but iced-water, which was prohibited on account of her pectoral affection. I found her with a short frequent cough, and when it increased much, she said it induced vomiting, but even when least pressed by the cough, she remarked that she had occasional calls to vomit. Her lips were blistered from the violence of the fever; her gums presented dark or purple spots of a few days' duration, and, though some of these appeared like superficial ulcers, there was no discharge of blood from them, as happens in cases of scurvy.

This woman was bled twice after my visit, once in each arm, and the relief it afforded her was such that she was heard to exclaim, "la sangria me ha dado la vida!"—the bleeding has given me life! Now the fever became intermitting, and she was cured in the ordinary way by the aid of sulphate of quinine.

(*To be continued.*)

